# Membrane protein trafficking in the anti-tumor immune response: work of endosomal-lysosomal system

**DOI:** 10.1186/s12935-022-02805-6

**Published:** 2022-12-17

**Authors:** Yan Jin, Zhifeng Deng, Ting Zhu

**Affiliations:** 1 Cancer Center, Renmin Hospital of Wuhan University, Wuhan, 430060 China; 2 Department of Otolaryngology Head and Neck Surgery, Renmin Hospital of Wuhan University, Wuhan, 430060 China

**Keywords:** Immune checkpoint, Anti-tumor immune, Endocytosis, Traffic, Recycle

## Abstract

Immunotherapy has changed the treatment landscape for multiple cancer types. In the recent decade, great progress has been made in immunotherapy, including immune checkpoint inhibitors, adoptive T-cell therapy, and cancer vaccines. ICIs work by reversing tumor-induced immunosuppression, resulting in robust activation of the immune system and lasting immune responses. Whereas, their clinical use faces several challenges, especially the low response rate in most patients. As an increasing number of studies have focused on membrane immune checkpoint protein trafficking and degradation, which interferes with response to immunotherapy, it is necessary to summarize the mechanism regulating those transmembrane domain proteins translocated into the cytoplasm and degraded via lysosome. In addition, other immune-related transmembrane domain proteins such as T-cell receptor and major histocompatibility are associated with neoantigen presentation. The endosomal-lysosomal system can also regulate TCR and neoantigen-MHC complexes on the membrane to affect the efficacy of adoptive T-cell therapy and cancer vaccines. In conclusion, we discuss the process of surface delivery, internalization, recycling, and degradation of immune checkpoint proteins, TCR, and neoantigen-MHC complexes on the endosomal-lysosomal system in biology for optimizing cancer immunotherapy.

## Introduction

Inhibitory immune checkpoints are expressed on tumor cells and T cells as membrane proteins and regulate T cell activation through costimulatory signals. For example, PD-L1 on tumor cells binds to PD-1 on T cells to inhibit the activation of T effector cells, thus inhibiting the immune response of tumors. The expression of PD-L1 is regulated by the process of internalization and reexpression. In addition to the PD-1/PD-L1 antibodies, the expression of PD-L1 on the surface of cancer cells can be regulated by the process of internalization, which affects immunotherapy [1]. For other immune checkpoint membrane proteins, CTLA4, LAG3, KIR, CD94/NKG2, and CD70 have the same process of internalization [2].

Preliminary results from clinical trials of cancer vaccines suggest that dendritic cells, peptides, and RNA-based neoantigen vaccines are safe and can induce CD8^+^ and CD4^+^ neoantigen-specific T cell responses [3]. A vaccine that induces a response to a tumor-derived neoantigen should induce a more robust immune response and cause less autoimmune-related toxicity than a vaccine based on tumor-associated antigen (TAA) [4]. Despite the promise of these early-stage cancer vaccine trials, the vast majority of neoantigen vaccine treatment results in clinical trials have been disappointing due to the low cross-presentation efficiency of antigen-presenting cells (APCs) [5], one of the leading causes of which is lysosomal neoantigen/adjuvant degradation.

In addition to immune checkpoint membrane proteins, preliminary studies suggested that the expression of immune-related transmembrane domain proteins such as T-cell receptor (TCR) and major histocompatibility (MHC) were also regulated by the endosomal-lysosomal system [6]. Immunotherapy can support tumor-specific T cell initiation to produce long-lasting anti-tumor responses. However, the expansion of tumor-specific T cells in vitro is limited by tumor antigens presented by MHC rather than surface antigens on tumor cells [7]. Synthetic chimeric antigen receptors bypass MHC restrictions and directly target specific cytotoxicity on the surface of malignant cells, but they are limited to extracellular surface targets on tumor cells called neoantigens [8]. Despite neoantigen expression, tumor cells can evade immune recognition by losing MHC-associated antigen presentation [9].

Furthermore, dying cancer cells can release antigens that are presented to primary T cells in secondary lymphoid organs to form TCR and neoantigen-MHC molecular complexes on the membrane and participate in the immune response along with the immune checkpoints[10]. This review aims to summarize the role of endosomal-lysosomal systems in cancer antigen recognition, presentation and immune activation and elucidate the relationship among tumorigenes, tumor treatment, and endosomal-lysosomal system.

## Overview of the endosomal-lysosomal system

### Biological process of the endosomal-lysosomal system

Clathrin-mediated endocytosis (CME) is an essential pathway, by which most transmembrane proteins are transported into the cell [11]. Membrane proteins or the receptors and their ligands through clathrin-coated vesicles or clathrin-independent endocytosis (CIE) are trafficked from the cell surface to the cytoplasm [12]. Once internalized, the membrane proteins are sorted and subsequently targeted to various organelles. There are simple mechanisms involving vesicle transport controlling the protein density of the cell surface in endosomal-lysosomal system that the internalized proteins are recycled back to the plasma membrane where the proteins are recycled, or selected to lysosomal compartments, where the proteins are degraded [13].

### Key proteins involved in the transport of endocytic vesicles

The cell plasma membrane is a dynamic structure in which membrane proteins can be internalized by invagination of the plasma membrane to form primary endocytic vesicles. These vesicles transport membrane proteins to the early endosomes (EEs) in the peripheral cytoplasm. Most of the coated pits and coated vesicles are receptor-mediated endocytosis. The outer skeleton of the coated vesicle is clathrin, and the inner is adaptor protein [14].

Membrane proteins are transported into the cell by clathrin-coated vesicles, which are present in all nucleated cells [15]. The transmembrane proteins are concentrated in clathrin-coated pits because they have special sequence motifs in their cytoplasmic domains that interact with elements of adaptor proteins [16]. Therefore, the membrane proteins with rapid internalization through clathrin-coated pits are based on protein–protein signal recognition [17]. Adaptor protein binding with the cytoplasmic domains of membrane proteins recruit clathrin into vesicles targeting various organelles [18]. Besides adaptor proteins and clathrin, the Rab proteins and SNAREs are playing key regulatory roles in involving in transport of endocytic vesicles.

Rab proteins are GTPase regulatory proteins, playing vital regulatory roles in most vesicular trafficking system among organelles [19]. Rab protein serves as a molecular switch in the regulation of vesicle transport [20]. Regulated by guanine nucleotide exchange factors and GTP-activated proteins, Rab protein switches between the active form of Rab-GTP and the inactive form of Rab-GDP. During the vesicle transport, Rab proteins interact with different downstream effector proteins, involving in the selection of cargos from the donor membrane, the formation of vesicles by budding, the regulation of vesicles movement along the cytoskeleton, and the anchoring and fusion of vesicles with the receptor membrane [21, 22]. For example, Rab4 regulates the fast recycling of cargo directly from EE and the trafficking from EE to the endocytic recycling compartment (ERC), while Rab11 regulates exit of slow recycling of cargo from ERC back to the plasma membrane. Endocytosis is also regulated by the C-terminal Eps15 Homology Domain (EHD) family of proteins, and EHD proteins, similar to the GTP-binding proteins of Ras-family, bind and hydrolyze ATP for their oligomerization and localization to tubular and vesicular membranes [23]. EHD1 is distributed to long tubular membranes and vesicles that generally emanate from the ERC. Molecule interacting with CasL (MICAL)-like 1 (MICAL-L1), as a novel EHD1 interaction partner, involved in the recruitment of EHD1 to tubular ERC membranes and regulates recycling.

Soluble N-ethyl maleimide sensitive factor-attachment protein receptor (SNAREs) are widely recognized as a vital element of the membrane fusion protein complex. The SNAREs regulate all fusion events of the membrane system. SNAREs can initiate vesicle fusion in the vesicle transport and participate in the exocytosis activity. SNAREs are originally divided into target membrane-associated SNAREs (T-SNAREs) and vesicle-associated SNAREs (V-SNAREs) according to their distribution locations [24]. The V-SNARE protein located on the vesicle membrane and the T-SNARE protein located on the target membrane pull the two membranes closer and drive the membranes fusion in the process of forming the SNARE complex [25]. N-ethyl-maleimide-sensitive factor (NSF) protein binds to the SNARE complex via adaptor soluble NSF attachment proteins (SNAP) to form the 20S complex, hydrolyzing ATP to provide energy to untangle the SNARE complex, depolymerizing it into monomers for recycling [26].

#### Early endosome

Once internalized, primary endocytic vesicles with molecules are transferred to the EEs marked by early endosome antigen 1 (EEA1) [27]. EE serves as the central sorting station in the endocytic pathway and the EE is known as sorting endosome [28]. Its function is providing an acidic microenvironment where the internalized receptor-ligand complex and other cargos are dissociated to reach the correct destination. The main sorting mechanism in EE is based on organelle geometry rather than the recognition of specific sorting motifs of the cargo proteins [16]. The fusion of GTP-derived primary endocytic vesicles with sorting endosome is partly regulated by Rab5 and SNAREs [29–31]. Rab5 has been localized to the EE and is one of the markers of EE. The prominent effect of Rab5 stablishes a specific membrane domain in the endosome to recruit various protein components [30]. Most EEs are usually located along the plasma membrane, and patrol the peripheral cytoplasm by movement along microtubules or move to the late endosomes (LEs), which are regulated by Rab5 [30].

There are three known destinations after endosome sorting: plasma membrane, endocytic recycling compartment (ERC) and LE. Besides the cargo proteins are directly transported back to the plasma membrane through the recycling endosome, and cargos could be also transported to the plasma membrane via ERC. The remaining molecules are then transported to LEs, further into lysosome. The surface of plasma membrane of the EE contains clathrin and component of the endosomal sorting complex for transport (ESCRT), whose protein is indirectly involved in the fusion, and interaction with homotypic fusion and vacuole protein sorting protein, which is responsible for sorting of ubiquitinated membrane proteins into intralumenal vesicles, which are formed by the membrane of EEs [32]. The ubiquitylation of the protein cytoplasmic domain is ultimately as a signal for targeting the protein to LEs or lysosomes to downregulation its express level via degradation [33]. Moreover, EE maybe communicates with the trans-Golgi network (TGN) by bidirectional vesicles exchange.

#### Late endosome

EE matures into LE when moving along the microtubules to the perinuclear region. And LE is also called multivesicular body because of the heterogeneous and variable size and composition, and most LEs have a multivesicular morphology. The naive LE grows in size by undergoing homotypic fusion reaction and acquiring additional lumen contents from intralumenal vesicles. In the process from EE to LE, Rab5 positive organelles are transformed to Rab7 positive, which marks the maturity of the LE [34]. In addition, the maturation of LE includes the exchange of membrane components, the movement to the perinuclear region, the transformation of fusion object, the formation of additional intralumenal vesicles, the decrease of intraluminal pH, the accumulation of lysosomal components and the change of morphology [35]. LE completes a significant transformation and has almost no similarities compared to EE.

LE acts as a secondary sorting station, and intralumenal vesicles and soluble lumen contents in LEs can be fused and degraded by lysosomes. If no incoming transport from endosome, lysosomes will lose integrity, acidity, and perinuclear localization. In addition to the direct delivery of proteins to lysosomes, LE also mediates the transport of lumen components from the TGN to lysosome. In addition to endocytosis and TGN-derived traffic, LE also works in crossroads with the autophagy pathway. The autophagosome fuses with LE or lysosome to acquire degradative capacity, and syntaxin 17(STX17), autophagy-related 14(ATG14), vesicle-associated membrane protein 8(VAMP8) are vital proteins in the membrane fusion of autophagosome and lysosome [36]. In some antigen-presenting cells, LEs fuse with the plasma membrane, squeezing their vesicular cargo into the extracellular space, known as the pathway of exosome secretion.

#### endo-lysosome

When the LE delivers the endocytic cargo to the lysosome, LEs fuse with classical dense lysosomes to form transient mixing organelles known as endo-lysosomes, where the degradation of cargo occurs. Like other fusion events in the membrane trafficking system, endosomal-lysosomal fusion conforms to the principles of the SNARE hypothesis. In addition, NSF and soluble NSF attachment proteins are required for membrane fusion, and their specificity is determined by SNAREs and Rab proteins. The SNARE for the fusion of LE with lysosome is identified in a cell-free system, which involves syntaxin 7, Vti1b, syntaxin 8, and vesicle-associated membrane protein 7 [37]. Endo-lysosome can mature to form classical dense lysosome, which involves the concentration of lumenal contents, removal of membrane proteins, and the recovery of SNAREs, ultimately working as a storage organelle for membrane components and hydrolases [35]. After endosomal-lysosomes are formed, some components may recycle back to cytoplasm through vesicle transport, such as mannose-6-phosphate receptors, tetraspanins, and SNAREs. Once a hydrolysis reaction triggers, the proteins are degraded catalyzed by proteases, lipases, nucleases, and other enzymes inside the lysosome, and these enzymes determine the total catabolic capacity of lysosome [38].

**Fig. 1:**
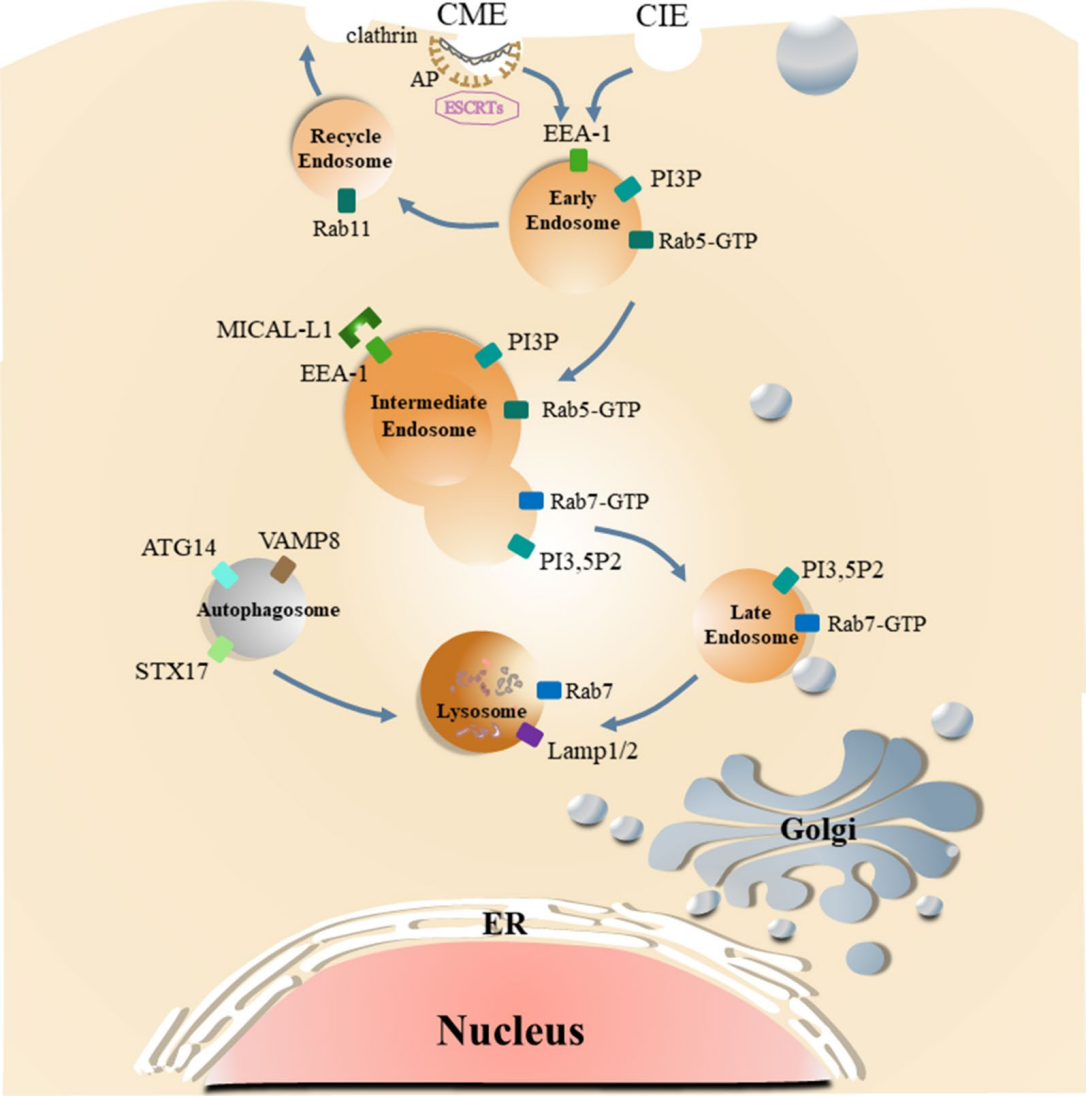
**Schematic diagram of the vesicles transport process of cargo in endosomal-lysosomal system.** Cargo is endocytosed from the cell surface in the form of vesicles with the interaction of the adaptor protein and ESCRTs to the cytoplasm via CME or CIE way. Most vesicles pass through early endosomes, intermediate endosomes and late endosomes via clathrin, and finally be degraded in lysosome, or are also recycled to the plasma membrane by recycle endosomes. Autophagosomes fuse with lysosomes to degrade cellular components, which is origin of autolysosome. Each organelle has its own marker proteins. EE is marked by EEA-1, PI3P and Rab5-GTP. Late endosome is marked by EEA-1, PI3, 5P2 and Rab7-GTP. Intermediate endosome is the fusion of EE and LE, containing the markers of them. Lysosome is marked by Rab7 and Lamp1/2. Autophagosome is marked by ATG14, VAMP8 and STX17

As is shown in the Fig. 1, EE, LE and lysosome work as a dynamic and adaptable system. The ambiguity and heterogeneity of the endosomal-lysosomal system are elusive because organelles undergo constant maturation, transformation, fusion and fission. It also explains the reason why there are lack of standard, universally agreed concepts and models of endosomal-lysosomal system.

## Checkpoint trafficking mediated by endosomal-lysosomal system

### PD-L1

In the case of cancers, tumors have overexpression of the PD-L1 to evade the immune system, which increases the possibility of tumorigenesis and invasiveness, making malignant cells less susceptible to specific CD8^+^ T cell-mediated lysis. Activated by the inflammatory factors, the expression of PD-L1 increases and plays an immunosuppressive function to help tumor cell escape from immune system attack. In addition, the transcription of PD-L1 can be controlled by many intracellular and extracellular signals through different pathways [39]. PD-L1 expression is regulated mainly by MAPK(RAS/RAF/MEK/ERK) and PI3K/Akt pathways. The PD-L1 format includes membrane PD-L1 (mPD-L1), cytoplasm PD-L1 (cPD-L1), nuclear PD-L1 (nPD-L1), serum PD-L1 (sPD-L1) and exosome PD-L1 (ePD-L1) [40]. PD-L1 degrades via proteasomes or lysosomes in different ways, leading to significantly enhanced cancer immunotherapy [1]. The ckLF-like MARVEL transmembrane domain contains 6 (CMTM6), is encoded by chromosome 3 and colocalizes with PD-L1 at the plasma membrane, regulating PD-L1 recycle back to cell surface [41]. Endosomal PD-L1 binds to CMTM6 to promote recycling and inhibit lysosomal ubiquitination and degradation. Tumor cells without CMTM6 show that PD-L1 recycling and surface-level reduced, leading to less inhibition of T cell activity [42]. Whereas H1A or STM108 (a PD-L1 antibody) abolishes the binding of PD-L1 to CMTM6, resulting in PD-L1 degradation by lysosomes [43]. Besides CMTM6, many proteins and chemicals have been identified with experimental confirmation to regulate the lysosomal degradation of PD-L1 (Fig. 1). Huntingtin-interacting protein1-related (HIP1R), PKCα/GSK3β/MITF, ADAM10/17, and other endosomal sorting signals influence PD-L1 autophagic degradation by trafficking to the lysosome, which is also inhibited by DHHC3 and Sigma I and the developing drugs [44–46] (Fig. 1). In response to specific anti-PD-L1 antibodies, PD-L1 protein is degraded via autophagy [1]. HIP1R is a intracellular regulator in PD-L1 lysosomal degradation. The functions of HIP1R relied on two sequence stretches—one involved in the interaction with PD-L1 and the other has a lysosomal sorting motif for targeting to the lysosome with the help of AP complex, and ALIX/ESCRT ubiquitin ligase [46]. SA-49 is a novel aloperine derivatives and decreased the expression of PD-L1 in NSCLC cells [47]. SA-49 suppresses GSK3β activity through PKCα-mediated mechanism inducing the MITF translocation and promoting the translocation of PD-L1 to lysosome for degradation [47]. Transport protein particle (TRAPP) is known as trafficking protein particle and multisubunit protein complex that regulates membrane trafficking by acting as guanine nucleotide-exchange factors [48]. Trafficking protein particle subunit 4 (TRAPPC4) maintains the expression of PD-L1 by recycling of PD-L1 mediated by Rab11 and protecting PD-L1 from lysosomal degradation [48]. Palmitoylation of PD-L1 inhibits the ubiquitination of PD-L1 and PD-L1 lysosomal degradation mediated by ESCRT [42]. TBM-1 triggers PD-L1 lysosomal degradation in a TFEB-dependent, autophagy-independent pathway [49]. Several drugs and proteins have been experimentally validated for use in antitumor therapy through the endosomal-lysosomal system. Endocytic inhibitors or autophagy inhibitors isolation of PD-L1 in endosomes or autophagosomes, cytoskeleton or STAT3 inhibitors disruption of clathrin-mediated endocytosis, and cell cycle inhibitors targeting protein-α/β molecule for nuclear importation participate in the trafficking of PD-L1 in antitumor theory. SA-49 is a novel aloperine derivatives as a new regulator of PD-L1 expression, and SA-49 decreased the expression of PD-L1 in NSCLC cells [47]. SA-49 promoted translocation of PD-L1 to lysosome for degradation. SA-49 acts PKCα and suppresses GSK3β activity inducing MITF translocation [47]. Sigma1 is a unique integral membrane chaperone or scaffolding protein in the secretory pathway and is enriched in the ER of cancer cells. Sigma-1 receptor (SIGMAR1) is associated to endoplasmic reticulum and cell surface. Disruption of S1R signaling using S1R antagonist such as IPAG leads first to unfolded protein response followed by autophagy [50]. Small-molecule Sigma1 modulators can be used to regulate PD-L1 in cancer cells and trigger its degradation by selective autophagy [44]. HIP1R binds to PD-L1 by its conserved C-terminal domain and targets PD-L1 to lysosomal degradation by an intrinsic sorting signal, PD-PALM is palmitoylation peptide targeting PD-L1 lysosomal degradation [46]. STM108 is a mouse antibody that recognizes human PD-L1. The results showed that STM108 mediated PD-L1 internalization to the lysosomes as indicated by the detection of red fluorescence when the pH was decreased from 7.0 to 4.5 [51]. H1A destabilizes PD-L1 by disrupting its binding with the PD-L1 stabilizer CMTM6, resulting in greater PD-L1 degradation through the lysosome [43]. verteporfin effectively caused the death of EGFR‐TKI‐resistant lung cancer cells by decreasing the expressions of p62 with oncogenic function, YAP, and its target PD‐L1[52]. As mentioned above is listed in the Table 1.

**Table 1:** The antitumor drugs targeting the PD-L1 trafficking

Drugs or therapy	Regulatory signal	References
SA-49	PKCα/GSK3β/MITF	[47]
TBM-1	TFEB	[49]
IPAG	Sigma 1	[44]
2-BP	DHHC3	[45]
PD-LYSO	HIP1R	[46]
STM108	EGFR/B3GNT3	[51]
H1A	CMTM6	[43]
Verteporfen	Palmitoylation, glycosylation	[52]
Curcumin	Palmitoylation, glycosylation\STAT\proteasome	[123]
Pitstop 2	Recycle	[124]
Ivermectin	Changes in redistribution, macromolecular interactions	[125]
Diformin	Isolation of PD-L1 in endosomes or autophagosomes	[126]
Amlodipine	Target PD-L1 to lysosome	[127]
LYTAC	Lysosomal targeting chimaera, extracellular proteins	[128]

### PD-1

**Fig. 2:**
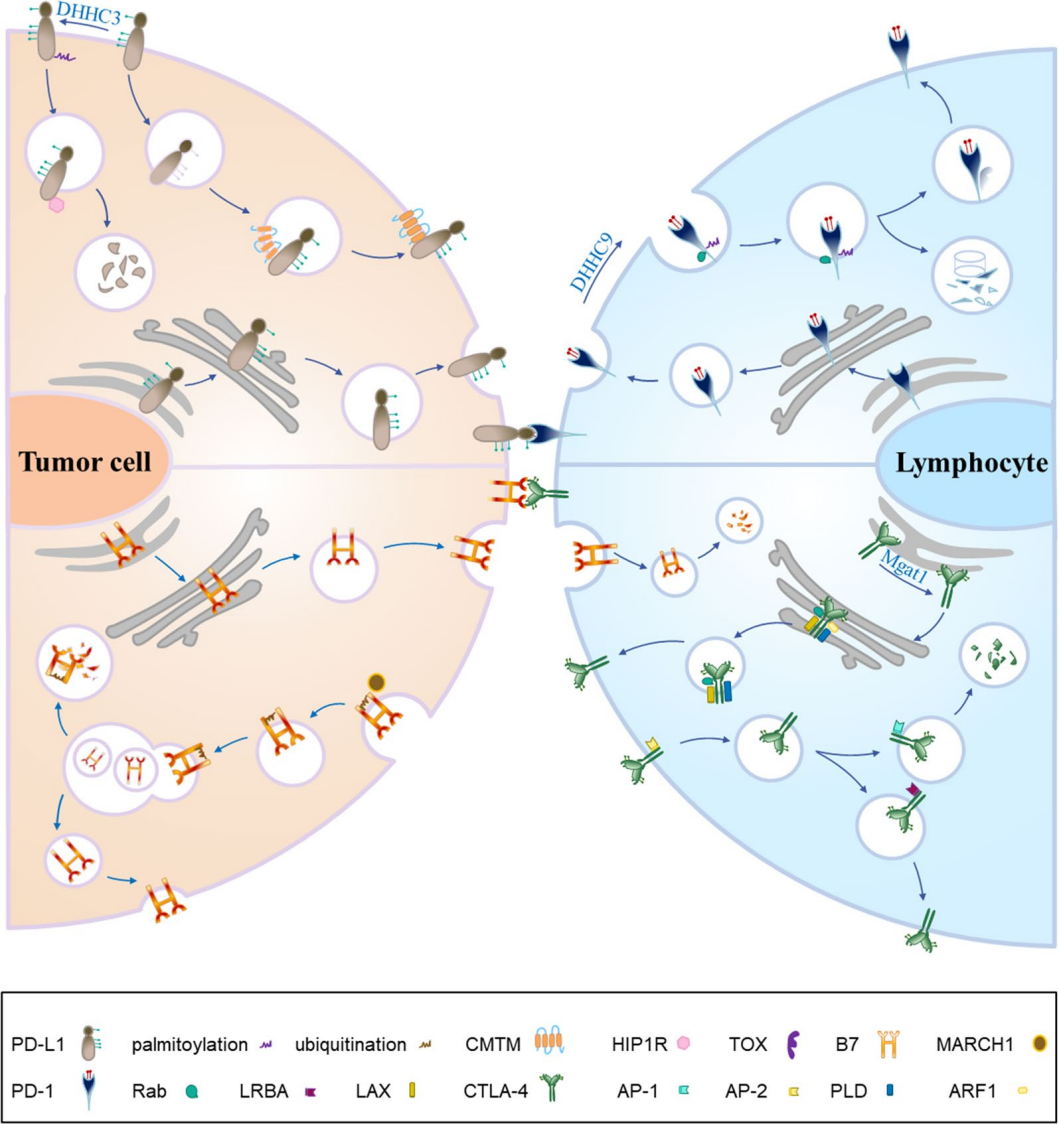
**The endocytosis, trafficking and degradation of immune checkpoint PD-L1 and B7(CD80/86) on the tumor cell and PD-1 and CTLA-4 on the lymphocyte.** ICs are transcribed and translated in the endoplasmic reticulum, modified in the Golgi apparatus, and vesicles are transported to the plasma membrane, where ICs interaction with the ligand. Some vital regulator controls the endocytosis, recycling and degradation of ICs. CMTM-family proteins promote the recycling of PD-L1 back to the plasma membrane and inhibits the lysosomal degradation of PD-L1; HIP1R mediates the lysosomal degradation of PD-L1; And DHHC3 palmitoylates the PD-L1 promoting the lysosomal degradation of PD-L1. The expression of the March-I down-regulating CD86. Rab11 and PD-1 co-locate in the recycle endosomes. DHHC9 palmitoylates the PD-1 promoting the lysosomal degradation of PD-1; On the contrary, TOX binds with PD-1 in the cytoplasm and facilitating PD-1 recycling. CTLA-4: Reduction of LRBA promotes the lysosomal degradation of CTLA-4

**Fig. 3:**
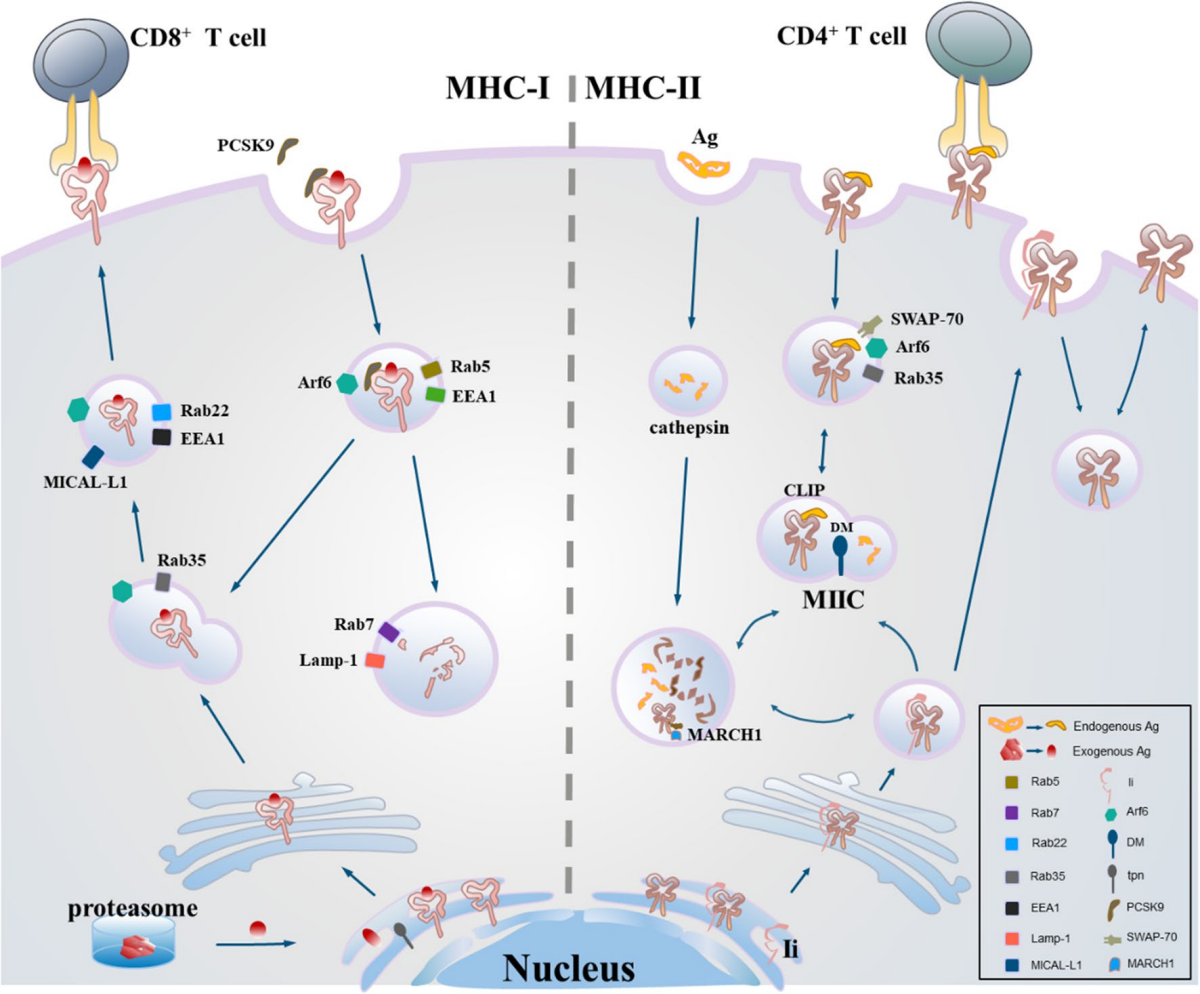
**The synthesis and antigen-presentation processes of MHC molecular.** MHC-I loads the polypeptide of endogenous antigens treated by proteasome in the endoplasmic reticulum under the action of TPN molecules; PCSK9 induced MHC-I to lysosome degradation; In the absence of PCSK9 cell, MHC-I is recycled to the cell surface after endocytosis in the clathrin-independent, Arf6-dependent pathways. MHC-II binds to the Ii chain in the endoplasmic reticulum, and Ii chain dissolves in the MIIC, then Ii-free MHC-II loads the exogenous antigens treated by lysosomes under the action of DM molecules; MARCH1 ubiquitinates β chain down-regulating the membrane MHC-II; SWAP-70 increases the membrane MHC-II through negatively regulating GTPases

PD-1 is a 55 kDa transmembrane protein of 288 amino acids belonging to the CD28 family of receptors [53, 54]. PD-1 contains an extracellular IgV-like domain that also is composed of a transmembrane domain as signal sequence, and a intracytoplasmic domain as immunoreceptor tyrosine-based inhibitory motif (ITIM) and immunoreceptor tyrosine-based switch motif (ITSM) [55] Figs. 2 and 3.

PD-1 is expressed by activated CD4^+^ and CD8^+^ T cells, B cells, dendritic cells (DCs), monocytes, and natural killer cells(NKs) [55]. Transcriptional factors or epigenetic factors can influence the transcription of PD-1. After translation, post-translational modifications such as fucosylation and N-glycosylation are crucial for PD-1 stability, and PD-1 stability can also be affected by miRNA. Once the stability of PD-1 is broken, it is degraded by proteasome and lysosome [56]. PD-1 has been localized in vesicles near the Golgi and TGN. These PD-1 vesicle reservoirs are waiting for TCR activation. Once PD-1 vesicles reach the plasma membrane, it can be ubiquitylated, triggering endocytosis and degradation [56]. Like PD-L1, the palmitoylation of PD-1 promotes the interaction between PD-1 and Rab11, promoting PD-1 transport to the recycling endosome and attenuating the degradation in the lysosome. Study showed the thymocyte selection-associated high mobility group box protein (Tox), a master transcription factor of T cell exhaustion, binds with PD-1 in the cytoplasm and facilitating PD-1 recycling [57]. 2-BP, a blockade of PD-1 palmitoylation, can disrupt the PD-1 and Rab11 interaction, subsequent enhancing anti-tumor immunotherapy [58].

### CTLA-4

Cytotoxic T lymphocyte-associated protein 4(CTLA-4) surface trafficking has recently gained a great attention in the field of tumor immunotherapy, and the precise regulatory mechanisms and vital regulatory sites of CTLA-4 are still emerging. CTLA-4, a glycoprotein in the immunoglobulin superfamily, has a similar structure to CD28. For human CTLA-4, there are three isotypes: the full-length isotype (flCTLA-4), ligand-independent form lacks the extracellular domain form (liCTLA-4), and the soluble form (sCTLA-4) lacks the exon encoding the transmembrane domain [59]. CTLA-4 is mainly located in TGN, endosomes and lysosome. Although even CTLA-4 at instant expression after T cell activation, only small amounts of CTLA-4 can be detected on the cell surface, which is sufficient to induce negative signaling in immunoreaction [60]. The regulatory mechanism of surface expression of CTLA-4 is crucial for anti-tumor immunity.

Surface expression of CTLA-4 is tightly regulated: intracellular CTLA-4 mainly accumulates in lysosomes and accumulate at the early stage of T cell activation [61].Once CTLA-4 is delivered to the plasma membrane then is constitutively internalized from clathrin-coated vesicles, after internalization into endosomes, CTLA-4 can be re-expressed on the plasma membrane or shuttled to lysosomal for degradation. CTLA-4 vesicles fusing with endosomes can be partial recycled to the plasma membrane and most to the lysosome for degradation, thus the membrane CTLA-4 keeps a low level [61]. In lysosomes, CTLA-4 degrades in a relatively short time if without any stimulation. Upon T cell activated, secreted vesicles containing CTLA-4 from lysosomes may move towards the TCR junction site, resulting in increased CTLA-4 on the plasma membrane [60].

Effective targeting of vesicles requires the receptor to contain specific internalized signals. Although CTLA-4 lacks the dileucine motif, it has the Gly-Val-Tyr-Val-Lys-Met (GVYVKM) motif for AP-1orAP-2, PI3K, SHP2 and PP2A binding [61]. Through a Tyrosine in the cytoplasmic domain of CTLA-4 binds to the μ2 subunit of AP-2 in the clathrin-associated endocytosis [62]. AP-1 mediates CTLA-4 shuttle from TGN to endosomes and lysosomes. CTLA-4 binds transmembrane adaptor TRIM in the TGN to promote the formation and transport of newly synthesized CTLA-4 vesicles with clathrin-coated to the plasma membrane.

Lipid kinases, but not PI3K, are involved in lysosomal sorting of CTLA-4 by promoting its internalisation and degradation. The treatment of cells with the lipid kinase inhibitor Wortmannin resulted in a higher surface and intracellular expression of CTLA-4.

CTLA-4 rich vesicles are more recovered to the plasma membrane with the lipid kinase inhibitor suggesting that lipid kinases are involved in the sorting of CTLA-4. CTLA-4 encounters LRBA in recycle endosomes, and its interaction with LRBA rescues CTLA-4 from the lysosomal degradation for removal of ligands and recycle back to plasma membrane [63] that CTLA-4 captures and delivers ligands for degradation, but does not degrade itself [61]. The secretion of cathepsin D and β-hexaminosidase are parallel to the increased membrane expression of CTLA-4 and lysosomal glycoprotein 85(a lysosomal marker), demonstrating that CTLA-4 membrane expression levels can also be enhanced by the secretion of CTLA-4 rich lysosomes [60]. CTLA-4 membrane expression also relates to guanosine triphosphatase (GTPase), adenosine diphosphate ribosylation factor-1 (ARF-1) and phospholipase D(PLD) function with the budding of vesicles from Golgi [61]. PLD inhibitors or dominant inactivation mutators of ARF-1 or PLD inhibit decrease the express of CTLA-4 on the plasma membrane.

### LAG3

Lymphocyte activation gene-3(LAG3) is the third inhibitory receptor exploited in human anti-cancer immunotherapy, behind PD-1 and CTLA-4. LAG3 belongs to the immunoglobulin superfamily, which is a 70 kDa transmembrane protein with four extracellular glycosylation sites, and is considered as a CD4 homolog, binds to MHC-II reducing cytokine and granzyme production while encouraging differentiation into T regulatory cells [64]. In addition to monomer form, part of LAG3 is expressed on the plasma membrane in dimer or oligomer form. Indeed, LAG3 is proteolytically cleaved by metalloproteinase ADAM10 and ADAM17, and solute LAG3 is most likely from proteolytic cleavage of membrane LAG3 rather than from the Golgi [65]. LAG3 surface expression is also regulated by endosomal-lysosomal system. Any molecules that disturb the trafficking from endosomal-lysosomal system to the plasma membrane have shown their unignorable significance in LAG3 trafficking as well [66]. Intracellular storage of LAG3 includes previously surface-expressed LAG3 that has been endocytosed and newly synthesized LAG3 waiting for rapid trafficking to the plasma membrane upon T cell activation. Approximately half of LAG3 is located in the LE and lysosome in unstimulated T cells and is rapidly translocated to the plasma membrane upon stimulation, ultimately degraded in the lysosome [67]. Endocytosis of LAG3 occurs following interaction with α-synuclein [68] but has not known about other LAG3 ligands FGL-1 and the lectins galectin-3 (Gal-3) and lymph node sinusoidal endothelial cell C-type lectin (LSECtin) [69]. LAG3 colocalizes with the symbol of endosome that GTPases, Rab5 and Rab7 and co-endocytoses with pathologic α-synuclein [68].

### KIR

Killing immunoglobulin-like receptor (KIR) is consisting of two or three extracellular C2-type immunoglobulin-like domains, a transmembrane portion and a cytoplasmic tail. KIRs have stimulant and inhibitory forms. The cytoplasmic tail of the inhibitory forms of KIR contains one or two immunoreceptor tyrosine inhibitory mods (ITIM), which play a part in inhibiting cell death by blocking FasL induction on stimulation to down-regulate immune responses, mainly targeting cell lysis and cytokine release [70]. Like MHC complexes, KIR has various polymorphisms and haplotypes, and their expression patterns on T cells is different clone. KIR was expressed on NK cells and T cell subsets, both CD4^+^ and CD8^+^T cells [71].

Both stimulant and inhibitory KIR bind to MHC-I on target cells. Studies have shown that the transport of both stimulant KIR and inhibitory KIR are regulated differently. And the recycling process of KIRs can be divided into two steps, endocytosis and exocytosis, where the amino acid sequences responsible to regulate are different. Data suggests that the phosphorylation of protein kinase Cs (PKCs) can up-regulate cellular KIR expression through stimulating the KIR maturation in endoplasmic reticulum–Golgi. In addition, the activate PKCs up-regulated membrane KIR levels by promoting the recycling of KIR through sorting endosomes. And KIR traffics to the plasma membrane through lytic granules in a PKCδ-dependent manner, and PKCδ promotes KIR is secreted to plasma membrane [72].

Stimulant KIRs have more unfamiliar with MHC-I than the inhibitory one, and they rely heavily on junction molecules, such as DNAX activating proteins DAP10 and DAP12, to deliver stimulus because they do not have immune-receptor tyrosine-based activation motifs (ITAMs) in their cytoplasmic mods. Study suggests that DAP12 knockdown is parallel with down-regulated membrane KIR level, so DAP12 contributes to the transport of KIR to the plasma membrane, because DAP12 facilitates the maturation of KIR through enhanced post-translational glycosylation to stabilize the KIR expressed on the plasma membrane and prevent the internalized KIRs from being degraded in lysosomes [73, 74]. At the same way, DAP10 can increase the transport of KIR to the plasma membrane and attenuate lysosome degradation.

### CD94/NKG2A

CD94 and NKG2A molecules can form heterodimers and homologous dimers, which are expressed on the surface of most freshly isolated NK cells and specific populations of CD8^+^T cells and CD4^+^T cells, but the expression levels are in great diversity. CD94/NKG2A is an inhibitory receptor and its ligand is HLA-E in humans, HLA-E is expressed by most normal cells, protecting themselves from NK cell aggression by interacting with CD94/NKG2A [75]. CD94/NKG2A is long-lived and continuously recycles back to the plasma membrane, maintained being constantly exposed to interaction with ligands. The trafficking of resilient membrane CD94/NKG2A begins with the co-endocytosed with fluid-phase markers, and the CD94/NKG2A endocytic vesicles in diameter (0.5–1.5 μm) seem to be much greater than micropinosomes (< 0.2 μm). Unlike the trafficking process of immune checkpoint above, known as an abbreviated intracellular trafficking pattern, CD94/NKG2A endocytic vesicles enter the EEs but do not enter LEs, nor do them fully enter the recycling compartment [76]. If this pathway turns out to be unique to CD94/NKG2A, the macropinocytic-like pathway indicates to be a process utilized by NK cells for its homeostasis [77].

### CD70

CD27, a transmembrane phosphoglycoprotein expressed on both CD4^+^ and CD8^+^ T cells, a member of the TNF receptor superfamily [78]. CD27 increases expression upon T-cell activation and shedding from the plasma membrane and formation of soluble CD27 (sCD27) upon activation. CD70 (CD27L) is a costimulatory receptor, the only ligand for CD27, which is a tightly regulated transmembrane glycoprotein primarily confined to activated lymphocytes and dendritic cells (DCs). CD70 is absent in resting cells and is transiently expressed at the cell surface of activate lymphocytes and APCs. In professional antigen-presenting cells, since the lack of intrinsic lysosomal targeting sequences in CD70, CD70 is delivered to the plasma membrane by default in cells without MHC-II presenting system. In cells with the machinery for antigen presentation by MHC-II, Ii coexpression directed it to LEs and lysosomes [79]. Introduction of MHC-II transactivator sufficed to reroute CD70 to MIIC. Vesicular trafficking of CD70 and MHC-II is coordinately regulated by the microtubule-associated dynein motor complex. When maturing DC contacts T cells, newly synthesized CD70 is specifically delivered via MIIC to the immunological synapse [79]. The trafficking of some immune checkpoints is shown in the Fig. 2.

## Immunological synapse and endosomal-lysosomal systems

In unstimulated T cells, the level of membrane TCR depends on the delicate balance of multiple processes, namely de novo synthesis and transport of newly assembled receptors, endocytosis of membrane TCR, recycling to the plasma membrane of internalized receptors or being degrading.

### TCR in the endosomal-lysosomal systemic circulation

The TCR is formed by an antigen-recognition module consisting of the α and β chains, and a signal-transducing module consisting of a ζ-chain homodimer and four clusters of differentiation 3 (CD3) chains present as γε and δε heterodimers. The intracellular domains of the ζ-chains and each of the CD3 chains contain ITAMs that allow for the recruitment of the intracellular signal transduction machinery upon TCR engagement of DCs [80]. The number of TCR-CD3 complexes is maintained by an equilibrium between the synthesis and secretion of new polypeptides. A major proportion of the TCR complexes is mobilized from endosome pool, undergoing delivery to the immune synapse membrane through microtubule-dependent recycling [81]. Since the rates of de novo synthesis and constitutive degradation of TCR are low, endosomal recycling is the principal mechanism exploited by T cells to regulate TCR expression [82, 83]. Additionally, the periodic transit of the TCR-CD3 complex inside the cell has been proposed as an opportunity of quality control of this long-lived receptor [84]. In the cell, internalized TCRs sustain signaling from an endosomal localization, and at the IS membrane, wherefrom miRNA-enriched exosomes and TCR-enriched ectosomes are released for transcellular communication with the cognate APC. With the IS, where engaged TCR-CD3 complexes concentrate and toward an endosomal CD3 compartment polarizes [85]. IS is mediated by an intracellular TCR pool associated with recycling endosomes that undergo delivery to the synaptic membrane to maintain a steady supply of receptors undergo activation-dependent internalization [86]. TCR-CD3 either to the recycling endosomes to be returned to the plasma membrane or the lysosomes for degradation. Regulators of vesicular trafficking, such as Rab35 and its GTPase-activating protein, the SNAREs, syntaxin-4, VAMP-3 and VAMP-7 and the adaptor UNC-119 are also recruited to the immune synapse, as a site of endosomal trafficking. These procedures rely on one of the most commonly used anti-CD3 antibodies, OKT3, which indirectly stimulates TCR-CD3 and mediates internalization and recycling [87]. The different subunits of the TCR-CD3 complex do not display the same intracellular trafficking dynamics. The involvement of regulating molecules in the intracellular fate of TCR-CD3, the location of sequences for internalization and intracellular sorting are still the main open questions.

### MHC trafficking in endosomal lysosomes

The expression of MHC on tumor cells is correlated with tumor cell survival and immunotherapy response intensity. However, the mechanism between the expression of MHC on the tumor cell surface associated with the immune response to tumor cells remains mysterious. However, with the deepening search of tumor immunity, more and more attention has been paid to the role of lymphocyte cells and MHC loading antigen peptides in tumor immunity [88].

#### MHC-I

Class I major histocompatible complex molecules (MHC-I) are trimers consisting of peptides with heavy chains bound in heavy chain grooves and light chain β_2_-microglobulin, and the whole complex is assembled in the endoplasmic reticulum and transported by the Golgi apparatus to the plasma membrane. In specialized APCs, peptides derived from extracellular antigens can also be loaded onto MHC-I through cross-presentation. The pathways in cross-presentation also presents antigens of vaccine and initiate tumor-specific CD8^+^T cell responses [89].

DCs may initiate different MHC-I transport pathways. Open conformer, fully load and suboptimal loaded MHC-I are internalized differently in the endosomal-lysosomal system. Inside non-specialist APCs, open and closed MHC-I are most likely to be dependent on Arf6 and clathrin-independent pathways with different dynamics to internalize and recycle [90].Study showed the open and closed MHC-I converges into Rab5-positive endosomes [89]. The CIE pathway, named after the GTPase Arf6, has been widely described as the endocytosis mechanism of MHC-I molecules. Study showed that the clathrin-independent, Arf6-dependent pathways are also involved in the recycling of integrin-β1, CD1, IL-2 receptors and CD59 adjacent to MHC-I [91]. Fully loaded molecules are transported into LEs and lysosomes, and most flow into recycling way. In addition, open conformational isomers may also be formed by dissociation of β_2_-microglobulin in the EE, which mature into LE and obtain LAMP-1 [92]. MHC-I can be recycled to the plasma membrane with EHD-1 and EHD-3 in a process regulated by Arf6 and Rab22 [93–95]. EHD1 co-locates with MHC-I in tubular recycle endosomes, and EHD1 overexpression enhances MHC-I recycling. In addition, there is also an outlet for a cyclic route for open former internalization and intracellular transport that can be achieved by ubiquitination of MHC-I [96]. The sorting motif in the tail of MHC-I may regulate sorting into and out of the Golgi apparatus [97]. LAMP-1 positive compartments are important for the loading of peptides with MHC-I by cross-presenting, and the transport of MHC-I through LE playing an important role in cross-presenting soluble proteins [97]. According to the speculative replacement model, MHC-I molecules are recycled or targeted for lysosomal degradation depends on the affinity of the binding peptide and association with β_2_-microglobulin [98]. Ubiquitination of the cytoplasmic tails of KSHV (Kaposis-sarcoma associated herpes virus) acting on the MHC-I molecule by inducing internalization to destroy epidermal growth factor receptors may be a sufficient signal to direct them to the lysosome for degradation[96]. Although peptide-loaded MHC-I can be recovered from EEs, most of the molecules are degraded in lysosomes once β_2_m is dissociated from the MHC-I heavy chain [92, 99, 100]. In specialized APCs, Rab11 and Rab22 modulate the presence of intracellular MHC-I reserves in a chamber similar to the endocytic circulation chamber (ERC), indicating that these molecules are derived from the plasma membrane [101, 102]. Moreover, the Arf6 mutant Q67L prevented the internalization and redistribution of MHC-I to TGN without affecting the MHC-I output to the surface [103]. In addition, Arf6-dependent endocytosis is cholesterol-dependent and blocked by filipin [104].

#### MHC-II

Class II major histocompatible complex molecules (MHC-II) are formed by two noncovalently associated chains, α and β, spanning the membrane; the α1 and the β1 domains together define the class II binding groove [105]. Direct Ii-associated MHC-II complexes (MHC-II-Ii) and Ii-free peptide-carrying MHC-II (pMHC-II) complexes shed from plasma membrane through completely different endocytosis and recycling mechanisms. Most MHC-II-Ii are connected to the plasma membrane from the TGN and then enters the endocytic pathway through clathrin-dependent endocytosis. Unlike MHC-II-Ii, pMHC-II endocytosis does not depend on the clathrin, AP-2, and dynamin [106].

The newly synthesized MHC-II is assembled with a chaperone protein in the endoplasmic reticulum, known as Invariant chain (Ii) with intracellular sorting signals that access some endocytic pathways and preventing antigenic peptides from binding MHC-II [107]. Rapid internalization of the MHC-II-Ii complex from the plasma membrane depends on dileucine-based sorting signals in the Ii cytoplasmic domain [108]. Mutations in these sorting signals prevent MHC-II-Ii from sorting into antigen processing region resulting in the accumulation of MHC-II-Ii complexes on the plasma membrane [109]. Antigenic peptides are usually produced by proteolysis of foreign proteins in the endosomal-lysosomal antigen processing region bound to MHC-II, defined as MHC class II–containing vesicular compartments (MIIC), where a large number of intracellular pMHC-II [110]. MHC-II can exchange antigenic peptides in the EEs in HLA-DM-dependent or HLA-DM-independent ways [111, 112]. MHC-II-Ii is directly transported from the TGN to this specialized MIIC, where Ii is proteolytic and degraded and with the help of the peptide editor HLA-DM, incoming antigenic peptides are loaded with Ii-free MHC-II forming pMHC-II [113]. Once loaded, the pMHC-II moves from the MIIC to the plasma membrane, presenting this pMHC-II to antigen-specific CD4^+^T cells [111]. In Hela-CIITA cells, pMHC-II is present in the LEs or lysosome with multivesicle, suggesting that the mechanism of regulating pMHC-II transport are conserved in the specialized APCs.

The transport of pMHC-II to the plasma membrane is regulated by mechanisms of endosomal-lysosomal system, by the small GTPases Rho and Rab through their regulation of the actin cytoskeleton, actin-based motor proteins and actin cytoskeleton rearrangement [114]. In activated DCs, surface localization of pMHC-II is achieved by direct fusion of MHC-II-containing vesicles with the plasma membrane, supported by tubular compartments extending from these endosomes to the cell membrane [115].Unlike the transport of Ii-associated MHC-II to the antigen processing region, most of the internalized MHC-II without Ii are rapidly recycled back to the plasma membrane [116].

Study showed lipopolysaccharide can increase the distribution of MHC-II in the plasma membrane [117]. Study showed SWAP-70 promotes surface localization of MHC-II in DCs through negatively regulating GTPases Rho, and a pleckstrin homology (PH) domain exists in the center region of SWAP-70, binding PIP3, the second messenger product generated by PI3K, and likely mediates membrane MHC-II localization [118]. Ubiquitination drives the endocytosis and sorting of MHC II to the luminal vesicles of multivesicular bodies (MVBs) one for lysosomal targeting degradation and the other for exosome secretion [119]. MARCH1, an E3 ubiquitin ligase, ubiquitinating β chain down-regulating the surface MHC-II [120]. Rosenberg’s team successfully identified MHC-II that could be recognized by autologous TILs from a patient with metastatic cholangiocarcinoma by sequencing. A molecule-restrictive neoantigen achieved a durable tumor response after receiving a reactive CD4 T cell transfusion [121]. Some of neoantigen epitopes with the MHC-II restriction has also been identified in other solid tumors [121]. In addition, among the immunogenic neoantigen epitopes screened from three different mouse models of melanoma, colon cancer and breast cancer, 80–90% of immunogenic neoantigen epitopes screened in mouse models of some cancers were recognized by CD4^+^T cells rather than CD8^+^T cells, suggesting that tumor-specific neoantigens may bind more easily to MHC- II than to more restrictive MHC-I [122]. Thus, MHC-II restricted neoantigens are promising immunotherapy targets.

## Prospect

This review concluded that the transport processes of several important membrane proteins with the endosomal-lysosomal system. The trafficking processes of membrane proteins after post-translational modification are tightly regulated on the protein level by endosomal-lysosomal system, involving surface delivery, internalization, recycling and degradation. The endosomal-lysosomal system not only associated with concentration and metabolism of drug, but also with the surface expression level of immune molecules, which are in relation to antitumor immunotherapy drug resistance. Through target regulation of endosomal-lysosomal systems to regulate surface expression of membrane proteins, combining with targeting receptor-ligand interaction drug or intracellular signal transduction drug may improve drug sensitivity and immunotherapy effectiveness. Immune checkpoints among these membrane proteins have been a hot spot in nearly a decade, and drugs targeting PD-1 and PD-L1 are under way in antitumor immunotherapy, as shown in the Table 1, the small molecule drugs reduce the degradation by reducing endocytosis and ubiquitination of PD-L1, thus increasing the expression of PD-L1 on tumor cell surface and with the PD-L1 monoclonal antibody targeting receptor-ligand interaction, improving the survival time of tumor patients. In summary, with the successful marketing of immune checkpoint blockers and the improvement of overall survival time of cancer patients with ICIs, tumor immunotherapies based on immune checkpoints has a bright future. However, although immune checkpoints related proteins in the immune microenvironment, antigen presentation related proteins in immune activation, tumor neoantigens and vital molecules in the formation of immune synapses are trafficked and degraded by endosomal-lysosomal system, different cargo seems to prefer different label molecules to perform their functions, and it is still poorly understood and studied in the regulatory proteins involved. In addition, the emergence of neoantigens makes it possible for tumor vaccines to achieve tumor cure, the research and application of neoantigens still need a long time to explore. Personally, I think tumor vaccine is the potential and most promising research target. Neoantigens can promote the immune system to work better to eliminate tumor cells and hit tumor cells accurately. Next, we need to expand the cellular and molecular biology of the endosomal-lysosomal system of immune molecules and test specific models and verify the hypothesis for the novel immunotherapy approaches into clinical. How the cargo is sorted and labelled how the internalized vesicles to anchor and how the intracellular regulatory molecules involve the transportation that remains to be further to investigated.

## Data Availability

Not applicable.

## Abbreviations

AP: Adaptor protein
ARF1: ADP ribosylation factor-1
ARF6: ADP ribosylation factor-6
ATG14: Autophagy-related 14
CIE: Clathrin-independent endocytosis
CME: Clathrin-dependent endocytosis
CMTM6: CKLF like MARVEL transmembrane domain containing 6
DHHC3: Aspartate-histidine-histidine-cysteine 3
DHHC9: Aspartate-histidine-histidine-cysteine 9
EE: Early endosome
EEA1: Early endosome antigen 1
EGFR: Epidermal Growth Factor Receptor
GSK3β: Glycogen synthase kinase-3β
HIR1P: Huntingtin interacting protein 1 related
ICIs: Immune checkpoint inhibitors
Lamp-1: Lysosomal associated membrane protein-1
LAX: Lysosomal associated membrane protein
LE: Late endosome
Ii: Invariant chain
IPAG: Labeled 1-(4-Iodophenyl)-3-(2-adamantyl)guanidine
LRBA: LPS responsive beige-like anchor protein
LYTAC: Target extracellular and membrane proteins for degradation
MARCH1: Membrane associated ring-CH-type finger 1
MICAL-L1: Molecule interacting with CasL like 1
MITF: Melanocyte inducing transcription factor
PI3P: Phosphatidylinositol 3-phosphate
PI3: Phosphatidylinositol-3
PKCα: Protein kinase C alpha
PLD: Phospholipase D
Rubicon: Lysosomal associated membrane protein
PCSK9: Proprotein convertase subtilisin/kexin type 9
Sigma 1: Sigma non-opioid intracellular receptor 1
STAT3: Signal transduction and transcription activator 3
STX17: Syntaxin 17
TGN: Trans Golgi network
TOX: Phosphatidylinositol-3
tpn: Tapasin
UVRAG: Ultraviolet resistance-associated
VAMP8: Vesicle-associated membrane protein 8
2-BP: 2-Bromopalmitate
5P2: 5-Bisphosphate
